# Decision Support for the Optimization of Provider Staffing for Hospital Emergency Departments with a Queue-Based Approach

**DOI:** 10.3390/jcm8122154

**Published:** 2019-12-05

**Authors:** Fuu-Cheng Jiang, Cheng-Min Shih, Yun-Ming Wang, Chao-Tung Yang, Yi-Ju Chiang, Cheng-Hung Lee

**Affiliations:** 1 Department of Computer Science, Tunghai University, No. 1727, Section 4, Taiwan Boulevard, Taichung 40704, Taiwan; admor@thu.edu.tw; 2 Department of Biological Science and Technology, National Chiao Tung University, No. 1001, Daxue Rd., Hsinchu 30010, Taiwan; 10chengmin@gmail.com (C.-M.S.); ymwang@mail.nctu.edu.tw (Y.-M.W.); 3 Information and Communication Research Division, National Chung-Shan Institute of Science & Technology, Taoyuan 32546, Taiwan; yjchiang0320@gmail.com; 4 Department of Orthopedics, Taichung Veterans General Hospital, No. 1650, Section 4, Taiwan Boulevard, Taichung 40705, Taiwan; leechenghung0115@gmail.com; 5 Department of Food Science and Technology, Hung Kuang University, No. 1018, Section 6, Taiwan Boulevard, Taichung 43302, Taiwan

**Keywords:** hospital emergency department, queuing theory, decision support, cost optimization

## Abstract

Deployment or distribution of valuable medical resources has emerged as an increasing challenge to hospital administrators and health policy makers. The hospital emergency department (HED) census and workload can be highly variable. Improvement of emergency services is an important stage in the development of the healthcare system and research on the optimal deployment of medical resources appears to be an important issue for HED long-term management. HED performance, in terms of patient flow and available resources, can be studied using the queue-based approach. The kernel point of this research is to approach the optimal cost on logistics using queuing theory. To model the proposed approach for a qualitative profile, a generic HED system is mapped into the M/M/R/N queue-based model, which assumes an R-server queuing system with Poisson arrivals, exponentially distributed service times and a system capacity of N. A comprehensive quantitative mathematical analysis on the cost pattern was done, while relevant simulations were also conducted to validate the proposed optimization model. The design illustration is presented in this paper to demonstrate the application scenario in a HED platform. Hence, the proposed approach provides a feasibly cost-oriented decision support framework to adapt a HED management requirement.

## 1. Introduction

### 1.1. Background

Hospitals play an important role in the healthcare system of society. They have changed rapidly in parallel with improvements in medical instruments and medicine. Administrators of hospital institutions should have spared no effort in developing strategies that enable the provision of high-quality services, while operating with increased costs and pressure from competition [1]. One of the most demanding departments in terms of economic resource consumption and programming is the hospital emergency department (HED). To this extent its operational profile should be monitored and optimized in order to provide the optimal quality of medical service subject to the budget constraint.

HEDs must be operational 24 h, and, moreover, should respond to multiple demands requiring sophisticated technical equipment and the manpower to operate them, all of which imply higher costs. Large HEDs have even higher costs because they offer a wide range of services that would be unavailable in a small rural HED [2]. Emergency Departments have traditionally been a crucial issue in the hospitals’ cost containment and management. The optimization of patient flow and bottleneck elimination in key departments could be a viable way for policy makers to decrease operational costs and boost quality of care [3]. In the interest of improving patient throughput and resource utilization, appropriate key performance measures are selected, like the size of staffing providers, HED patient arrival patterns, service rate of staffing providers, waiting time, etc. To explore the tradeoff among them, the proposed queue-based optimization technique on cost may provide hospital management with a decision support for deploying staffing providers under the constraints of the kernel performance parameters.

An M/M/R/N queuing model was adopted to explore the cost profile from analyzing the relationships among relevant performance parameters in a HED, such as the number of staffing providers (i.e., servers) needed during each staffing interval. This model assumes a single queue with a limited system capacity of N that feeds into R identical medical servers (i.e., staffing providers). The fourth symbol “N” of the notation M/M/R/N indicates the restriction on the system capacity of the HED. The value of (N−R) gives the number of waiting rooms for incoming patients when all staffing providers in the HED are busy. Arrivals occur according to a time-homogeneous Poisson process with a constant rate, and the service duration (e.g., provider time associated with a patient) has an exponential distribution. In the language of queuing theory [4,5], these two assumptions are often called Markovian, hence the use of the two ‘‘M’s’’ in the notation used for the model. One advantage of adopting the M/M/R/N model is that given an arrival rate, an average service duration, and the number of servers, formulae for performance measures such as the cost profile, the average number of patients, or the average waiting time can be easily obtained.

### 1.2. Contribution Profile

This novel idea in this work originated from the theory of an M/M/R/N queuing system (QS), which is used to estimate the optimal number of providers needed during each staffing interval [4,5]. At some pre-configured period (say a shift, or a day), a finite quantity of staffing providers exists to provide medical services for patients under limited waiting rooms in HEDs. On application modeling, such a finite quantity of staffing providers (i.e., R) can be regarded as the term “server” in the M/M/R/N model of queuing theory. The quantity of (N−R) can be considered to be the rather limited waiting rooms in HEDs regulated by each hospital.

The research goal for this work is to explore whether, with cost-based deployment, how many sets of staffing providers in the HED schedule would be optimal if a certain level of the server availability is kept? To explore the tradeoff between them, the proposed optimization technique may provide the HED management with decision support on the number of staffing providers. The key contributions of this paper are threefold: (1) This work provides HED administrators with an efficient deployment of staffing providers for the HED platform to optimize the cost improvement. In regards to management, the proposed system can be adopted as a decision-making methodology approaching predictive management, rather than reactive or chaotic management. (2) For quantitative analysis, the M/M/R/N queue model was applied and derived, and then the relevant system metrics were established in a brand-new manner. The mathematical expression of the cost function was established for the evaluation requirement. (3) In regards to verification, relevant experimental results were obtained in terms of configurations on cost optimization and average waiting time. The simulated results indicate that the proposed approach may provide a feasible decision support for deployment on quantities of standby servers.

The rest of the paper is organized as follows: Section 2 describes related work and the motivation behind this research. To demonstrate the framework qualitatively, an M/M/R/N model of queuing theory is adopted and the mapping profile is demonstrated in Section 3. Quantitative work is presented in Section 4, where the mathematical analysis is conducted in detail and the relevant system performance measures, such as the expected number of online servers, the expected number of spares, etc., are derived. Following this, in Section 5, the queue-based model is further addressed in terms of cost function, and the simulations of the feasibility of the proposed scheme are conducted. Finally, some concluding remarks are made in Section 6.

## 2. Related Work

HED crowding represents an important issue that may affect the quality and access of health care. Accordingly, the optimization of average waiting times has become a focus across many mainstream hospitals. As defined by the Canadian Association of Emergency Physicians [6], HED overcrowding is a situation in which the demand for services exceeds the ability of health care professionals to provide care within a reasonable length of time. As stated in [7], significant variation in HED patient arrival rates necessitates the adjustment of staffing patterns to optimize the timely care of patients. Green et al. [7] collected detailed HED arrival data from an urban hospital and used a queue-based analysis to gain insights on how to change provider staffing to decrease the proportion of patients who leave without being seen. However, no optimization materials in terms of mathematical theory were addressed at all in these studies [8,9,10].

Finamore et al. [6] described an innovative use of a satellite clinic to prevent patients returning to HED for care on a scheduled basis. Their strategy allows patients returning for follow-up diagnostics or treatment to bypass the main HED. The proposed HED satellite clinic may shorten the waiting times in multiple ways, such as increasing the capacity to remove returning patients from the pool of patients requiring care in the HED, and creating a separate registration area and a separated staffed treatment area. The visit data in the HED were used to measure crowding and completion of waiting room time, treatment time, and boarding time for all patients treated and released or admitted to a single HED during 2010. In [11], the authors conducted a relevant statistical analysis and concluded that a HED census at arrival demonstrated variation in crowding exposure over time. In the work of Wiler et al. [12], the authors developed an agent-based simulation model for the evaluation of the FTS (fast track strategies) scheme applied in the HED to reduce patient waiting time. By and large, the issues regarding cost optimization on the HED management cost are not a concern for these open studies [5,11,12].

Vass and Szabo [13] evaluated 2195 questionnaires in the HED situated in Mures County, Romania, for a period three years (2010–2013). Their research reported that long waiting times were the most important complaint in patient’s satisfaction surveys. To perceive the waiting times, only a specific M/M/3 queuing model was considered in their work to demonstrate the computation details. The work of [13] has motivated us to consider whether it is possible to provide an effective and feasible approach to decision support for the optimization of provider staffing under cost constraints for the HEDs with more elaborative queue-based frameworks. This research generalizes the queuing model of [13] into the M/M/R/N queuing framework in terms of three practical aspects: (1) Numbers of medical servers (provider staffing) can be configured to one of the system parameters instead of a fixed quantity. Such a dynamic staffing level enables a hospital to quantify the cost patterns and the alleviation of HED waiting times. (2) The space available in the HED would be limited for every hospital management. The fourth factor (N) in the notation of the M/M/R/N model symbolizes the fact that only N patients can be allowed to enter the waiting rooms of the HED in order not to exacerbate the issue of overcrowding. (3) The exact mathematical expressions would be derived in an elaborative manner and the relevant cost formulation would be used to provide the generic decision support for the hospital administrators.

## 3. The Proposed Model of Medical Emergency Services

### 3.1. The Generic Platform of Medical Emergency Services

This research explores feasible decision support that is proposed to optimize running costs under the constraint of the waiting time at a HED using queue-based models. The exemplified HED is in a metropolitan hospital (Taichung Veterans General Hospital or TVGH) located in central Taiwan. It began offering medical services on 16 September 1982. Since 1991, it has been accredited as a “Medical Center and First-Class Teaching Hospital” by the Department of Health, Taiwan. Taichung Veterans General Hospital is a 1500-bed hospital with up to 3900 employees. According to the latest statistical average data of registration accessed in TVGH, it offers a capacity of about 7000 outpatients and 190 patients in the emergency room daily [14]. As a public medical center, it provides safe, high-quality medical services with advanced facilities and training programs, as well as outstanding research and development programs.

**Figure 1:**
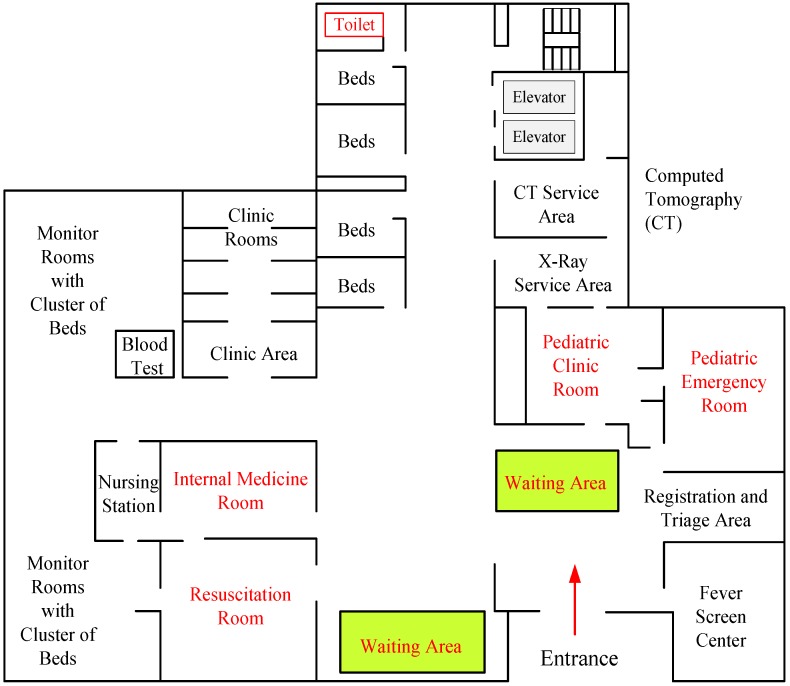
The functional deployment on the ground floor of the TVGH-ED building. TVGH-ED, Taichung Veterans General Hospital - Emergency Department.

The HED building, with eight floors at the TVGH (TVGH-ED), provides comprehensive emergency services 24 h a day. The functional deployment on the ground floor of the TVGH-ED building, as shown in Figure 1, is composed of different zones, including the Registration and Triage Area, Resuscitation Areas, Internal Medicine Areas, the Pediatric Treatment Area, Waiting Areas, Clinic Areas, Monitor Rooms and the Fever Screen Center, and relevant auxiliary service units such as the X-ray service and nursing stations. As this HED is a rather complicated service system due to random arrivals, various disease chains, uncertain service times of care, and randomness in human decision-making, it is difficult to model the whole HED with a single operational model. From the perspective of the model attribute [15], a generic operational model is defined as a formal description of operations performed to deliver a health service that is applicable over a wide range of health service delivery settings. For the sake of simplicity, this research concentrates the optimization issue on a specific platform of medical service, which is used hereafter to model staffing providers for a single disease chain.

### 3.2. Mapping Profile between the HED Service Platform and the M/M/R/N Queuing System

**Figure 2:**
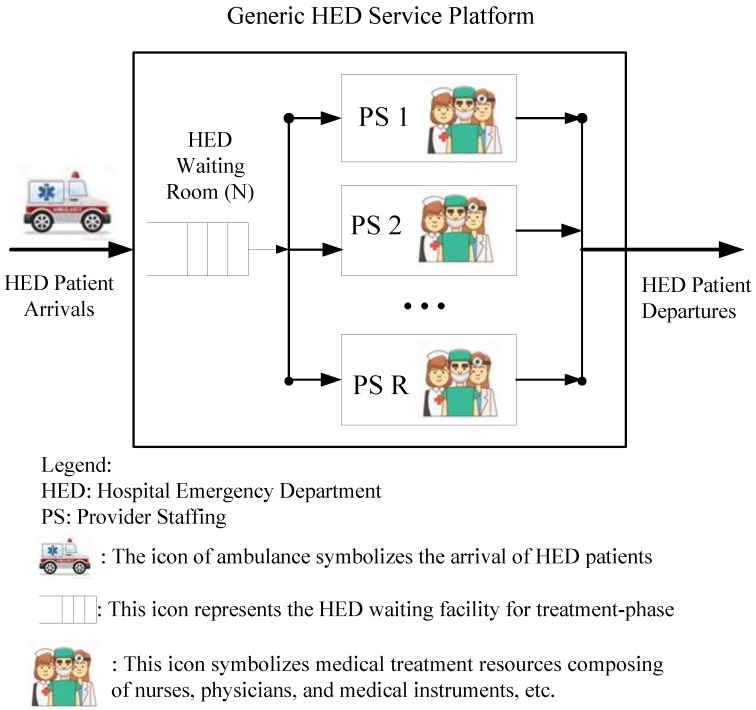
The generic service platform of an emergency department.

The proposed generic framework on the HED service platform is considered to be modeled as an M/M/R/N queuing system (QS), which is used to estimate the optimal number of providers needed during each staffing interval. An input-throughput-output framework of HED operations is used as the prototype shown in Figure 2 for a generic profile [16]. The ambulance icon symbolizes the arrivals of HED patients. Practically, patient arrivals are hard to schedule, or even control significantly. Arrivals may surge in some unpredictable time windows due to a short-term disaster, car accidents, and seasonal influences [17]. In modeling language, the busy and regular time windows can be associated with high and normal arrival rates, respectively. Patient arrivals in the proposed model are assumed to be Poisson processes [18], with average hourly rates that are forecasted for each future hour in question (say a shift, or a day) [19].

**Figure 3:**
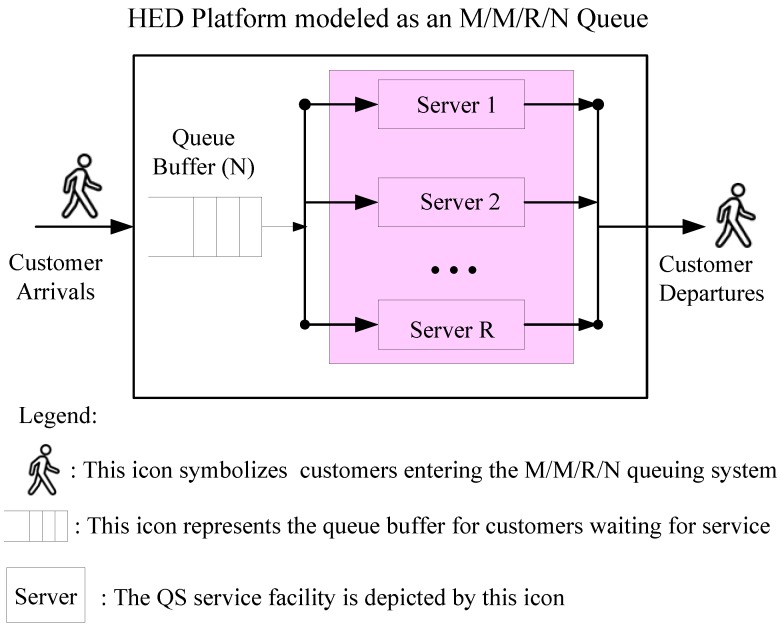
An M/M/R/N queue system mapped by the HED service platform.

The itinerary for HED patients from arrival to exit can conceptually be divided into three phases [12]. The first phase, named “Waiting for treatment phase (*waiting phase*)”, is symbolized by the icon HED Waiting Rooms in Figure 2. In the waiting phase, the patient goes through some standard processes that assist the HED to grasp the record of patients and their current medical condition. These are termed the Registration and Triage process, respectively. Registration guarantees administratively that patient demographics are captured accurately for billing and maintaining the record. Triage is the first assessment conducted by a healthcare professional after the patient arrives in the HED. The second phase (*treatment phase*) begins when the patient is placed in bed. For simplicity, the treatment phase is represented by the icons of provider staffing (medical servers) in Figure 3 for a generic profile. The whole medical service largely depends on patient acuity and physician activities. In modeling language, the duration of treatment can be regarded mathematically as the service rate of (medical) servers. The treatment phase is followed by the post-treatment phase, which is represented by the expression HED patient departures in Figure 3. Exiting from the treatment area of the HED is reasonably assumed to mean that the patients are discharged, either as an outpatient or into the hospital [16].

The mapping scenarios for the theoretical approach are illustrated in Figure 2 and Figure 3. An M/M/R/N queuing model was used to estimate the number of providers needed during each staffing time window. In Figure 3, the proposed model assumes a single queue with regulated and finite waiting rooms that feed into R identical servers with blue highlights, which is mapped to providers in the HED. The walking-man (customers) icons symbolize HED patient arrivals. Based on the proposed queuing model, relevant system metrics, such as average waiting times, expected number of customers in the queue buffer, and the probability that all servers are busy, can be analyzed and derived mathematically [7]. For instance, a patient’s total length-of-stay from arrival to departure from the HED platform is termed as the patient throughput time, which is equivalent to the waiting time in the QS. Patient throughput time has a significant impact on operational and economic efficiency as well as overall patient satisfaction, which is a measure of medical service quality [20].

Generally, the performance metric on average waiting times may provide the HED administrator with decision support on how to alleviate patients’ complaints. To avoid the deterioration of average patient throughput time (i.e., the average wait times in the QS), the optimization approach on the average waiting times, under some constraints such as a limited number of servers in the QS (i.e., mapped counterpart: level of staffing in the HED platform), is explored further in this article. The metric is the probability that all servers can be used to reveal the possibility and scenario in which notorious HED crowding may occur. The question is how to reduce this HED crowding phenomenon in some specific time windows. Such a metric can provide decision support for the administrator in order to properly configure or deploy hospital resources.

## 4. Quantitative Modeling and System Measures for the HED Platform

### 4.1. Theoretical Analysis

The service-oriented model on the HED platform in Figure 3 is considered to have R servers with an adequate level of staffing and a finite size (N) of waiting rooms for HED arrivals. The birth-and-death process is adopted to derive analytic steady-state solutions to the M/M/R/N queuing system (QS). Let the states n (*n* = 0, 1, 2, …, N) represent the number of customers in the QS. McManus et al. [18] studied all admissions to the medical–surgical intensive care unit (ICU) of a large, urban children’s hospital during a 2-year period. Their statistical analysis confirmed that the arrival rate of patients to ICUs follows a Poisson distribution, and the durations of stay (service times) were found to follow an exponential distribution. Hence, it is reasonably assumed that the customers arrive according to a Poisson process with mean arrival rate λ_n_ = λ if 0 ≤ n ≤ N and λ_n_ = 0 if n > N due to a finite system capacity. The QS has R servers, each having an exponential distribution of service times with an identical service rate µ_n_ = µ. The service volume can be classified into two parts as follows:

Mean Service Rate:

(1) $$\mu_{n}=\{\begin{array}{c} n\;\mu ,\;\mathrm{if}\;1\leq n\leq R\;\\ R\mu ,\;\mathrm{if}\;\left(R+1\right)\leq n\leq N \end{array}$$

**Figure 4:**
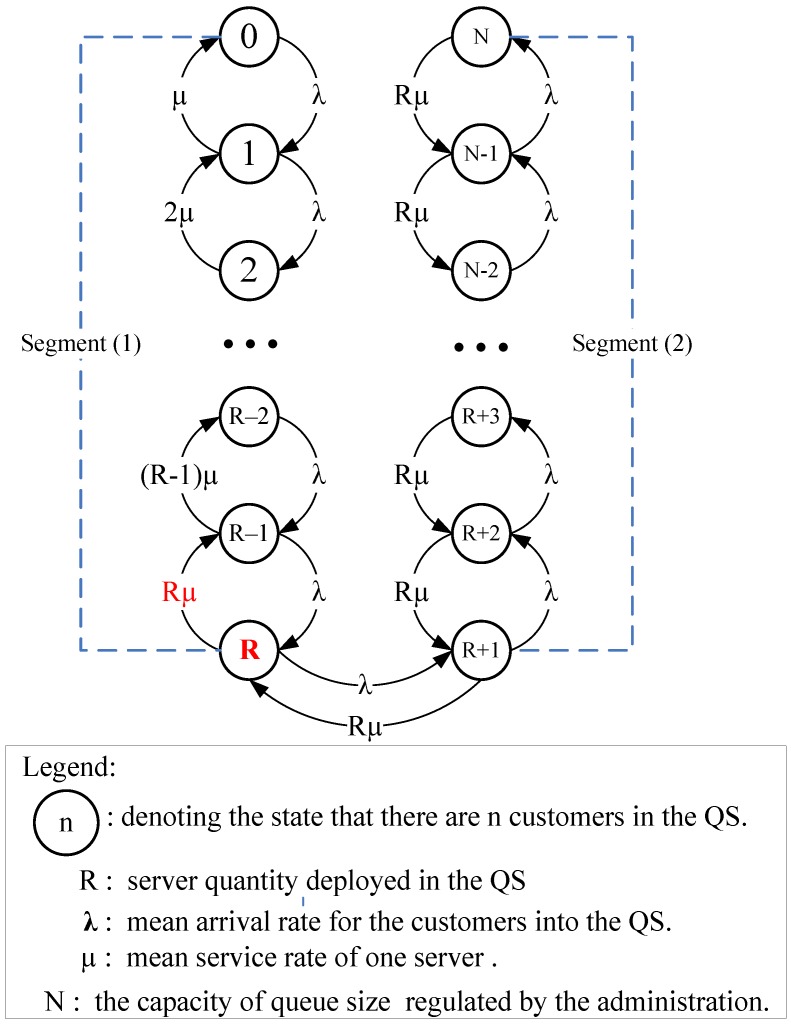
State-transition-rate diagram for the proposed model.

To approach analytic steady-state results for the proposed model, we first construct the state-transition-rate diagram depicted in Figure 4. The number inside the circle represents the number of customers (patients) in the system. Each circle in Figure 4 shows the steady-state probability scenario that may occur during the service period in the system. For each circle except the first one (n = 0) and the last one (n = N), there are four arrows marked with the corresponding values of the state-transition rate. The quantity marked along each arrow gives either the flow-in probability into that state or the flow-out probability off that state.

Let the notation P(n) = the probability that there are n customers in the system, where *n* = 0, 1, 2, …, N. Hence, for a steady-state case, the state probability functions P(n) can be obtained from the birth-and-death formula [5] in association with the state-transition-rate diagram shown in Figure 4. We define notation ρ = λ/µ for the server utilization and ρ_S_ = ρ/R = λ/(Rµ) for the system utilization. According to the value n (number of customers in the QS that may be present), two segments are defined by the vector: (Segment 1, Segment 2) = (1 ≤ *n* ≤ R, (R+1) ≤ *n* ≤ N). The state probability functions P(n) can then be derived in terms of two segments as follows:

(A) Segment (1): 1 ≤ n ≤ R
  (2) $$P\left(n\right)=\frac{\lambda_{0}\cdot \lambda_{1}\cdot \lambda_{2}\;\cdots \;\lambda_{n-1}}{\mu_{1}\cdot \mu_{2}\cdot \mu_{3}\;\cdots \;\mu_{n}}P\left(0\right)=\frac{\lambda^{n}}{\mu \;\left(2\mu \right)\left(3\mu \right)\;\cdots \;\left(n\mu \right)}P\left(0\right)=\frac{\lambda^{n}}{\mu^{n\;}n!}P\left(0\right)=\frac{\rho^{n}}{n!}P\left(0\right)$$

(B) Segment (2): (R+1) ≤ n ≤ N,
  (3) $$\begin{array}{c} P\left(n\right)=\frac{\lambda_{0}\cdot \lambda_{1}\cdot \lambda_{2}\;\cdots \;\lambda_{n-1}}{\left(\mu_{1}\cdot \mu_{2}\;\cdots \;\mu_{R})(\mu_{R+1}\;\cdots \;\mu_{n}\right)}P\left(0\right)=\frac{\lambda^{n}}{\left[\mu \cdot \left(2\mu \right)\cdots \left(R\mu \right)\right]\left(R\mu \cdots R\mu \right)}P\left(0\right)=\\ =\frac{\lambda^{n}}{R!\;\mu^{R}\;{\left(R\mu \right)}^{n-R}}P\left(0\right)=\frac{\rho^{n}}{R!\;{\left(R\right)}^{n-R}}P\left(0\right) \end{array}$$

There are (n–R) terms of Rμ in the parenthesis (Rμ. Rμ) of the above denominator. Equations (2) and (3) are the closed-forms for the state probability functions P(n) in terms of two segments in which the number of customers may be present. To obtain P(0), we substitute expressions (2) and (3) in the normalizing equation $\sum_{n=0}^{N}P\left(n\right)=1$, which yields:

$$\sum_{n=0}^{R}\frac{\rho^{n}}{n!}P\left(0\right)+\sum_{n=R+1}^{N}\left(\frac{\rho^{n}}{R!{\;R}^{n-R}}\right)P\left(0\right)=1$$

(4) $$P\left(0\right)={\left[\sum_{n=0}^{R}\frac{\rho^{n}}{n!}+\sum_{n=R+1}^{N}\left(\frac{\rho^{n}}{R!{\;R}^{n-R}}\right)\right]}^{-1}={\left[\sum_{n=0}^{R}\frac{\rho^{n}}{n!}+\frac{\rho^{R}\left(1-\rho_{s}^{N-R+1}\right)}{R!\;\left(1-\rho_{s}\right)}\right]}^{-1}$$

### 4.2. System Performance Measures

Mathematical expectations are crucially important for the long-run theoretical average values of relevant parameters in the system. To formulate the expressions of the system performance metrics, it is necessary to construct average-based functions, such as the expected number of customers in the queue, expected number of busy servers in the system, etc. The following mathematical analyses are all necessary for the system performance measures of an M/M/R/N QS.

Let

Ls = expected number of customers in the system,
Lq = expected number of customers in the queue buffer,
E[I] = expected number of idle servers,
E[B] = expected number of busy servers,
P_B_ = Probability that all servers are busy,
Ws = average waiting times in the system,
Wq = average waiting times in the queue buffer.

With steady-state probability functions (2) and (3), it yields

(5) $$L_{s}=\sum_{n=0}^{N}n\;P\left(n\right)$$

(6) $$L_{q}=\sum_{n=R}^{N}\left(n-R\right)\;P\left(n\right)$$

(7) $$E\left[I\right]=\sum_{n=0}^{R-1}\;\left(R-n\right)\;P\left(n\right)$$

E[B] = R − E[I](8)

(9) $$P_{B}=\sum_{n=R}^{N}P\left(n\right)$$

To express the above parameters in terms of (R, N, ρ, ρ_S_, P_0_), the system performance measures can be derived as follows:

(10) $$\begin{array}{c} L_{s}=\sum_{n=0}^{N}n\;P\left(n\right)=\sum_{n=0}^{R-1}\;n\;P\left(n\right)+\sum_{n=R}^{N}\;n\;P\left(n\right)=\sum_{n=0}^{R-1}\;n\cdot \frac{\rho^{n}}{n!}P\left(0\right)+\sum_{n=R}^{N}\;(n-R+R)P\left(n\right)=\\ =\sum_{n=0}^{R-1}n\cdot \frac{\rho^{n}}{n!}P\left(0\right)+\sum_{n=R}^{N}(n-R)P(n)+R\sum_{n=R}^{N}P(n)=\sum_{n=0}^{R-1}n\cdot \frac{\rho^{n}}{n!}P\left(0\right)+L_{q}+R\;P_{B} \end{array}$$

(11) $$P_{B}=\sum_{n=R}^{N}P\left(n\right)=\sum_{n=R}^{N}\;\frac{\rho^{n}}{R!{\;R}^{n-R}}P\left(0\right)=\frac{\rho^{R}}{R!}\frac{\;\left[1-{\left(\rho_{s}\right)}^{N-R+1}\right]}{\left(1-\rho_{s}\right)}P\left(0\right)$$

By changing the indices of **j** = **n**—R so that n = R is changed to j = 0, and n = N is changed to j = N—R,

(12) $$L_{q}=\sum_{n=R}^{N}\;\left(n-R\right)\;P\left(n\right)=\sum_{n=R}^{N}\;\left(n-R\right)\frac{\rho^{n}}{R!{\;R}^{n-R}}P\left(0\right)=\frac{\rho^{R}P\left(0\right)}{R!}\sum_{j=0}^{N-R}\;\left[j\cdot {\left(\rho_{s}\right)}^{j}\right]$$

The average waiting times in the system and in the queue buffer (Ws, Wq) can be derived by applying Little’s formula, which gives $W_{s}=\frac{L_{s}}{\lambda }$ and $W_{q}=\frac{L_{q}}{\;\lambda }$, respectively.

### 4.3. An Illustrative Example with Computation Details

To gain prompt perception on the theoretical implication of the quantitative modeling, an example is given by a detailed calculation. Let (R, N) = (4, 5) and (λ, μ) = (2, 1), then the server utilization ρ = λ/μ= 2 and the system utilization ρ_S_ = ρ/R= 0.5, which is less than unity for the stable system.

(1) 0 ≤ n ≤ (R–1) = 3, $P\left(n\right)=\frac{\rho^{n}}{n!}P\left(0\right)=\frac{2^{n}}{n!}P\left(0\right)$
  (13) $$P\left(1\right)=\frac{2^{1}}{1!}P\left(0\right)=2P\left(0\right);\;P\left(2\right)=\frac{2^{2}}{2!}P\left(0\right)=2P\left(0\right);\;P\left(3\right)=\frac{2^{3}}{3!}P\left(0\right)=\left(1.33\right)P\left(0\right)$$

(2) R ≤ n ≤ N, i.e., For 4 ≤ n ≤ 5, $P\left(n\right)=\frac{\rho^{n}}{R!{\;R}^{n-R}}\;P\left(0\right)$
  (14) $$P\left(4\right)=\frac{2^{4}}{4!\;4^{4-4}}\;P\left(0\right)=0.667\;P\left(0\right);\;P\left(5\right)=\frac{2^{5}}{4!\;4^{5-4}}\;P\left(0\right)=0.333\;P\left(0\right)$$
  ⇒[P(1), P(2), P(3), P(4), P(5)] = = [2, 2, 1.333, 0.667, 0.333] P(0).(15)

The complete five state probabilities are assembled and expressed in terms of P(0) as follows:

Using the normalization condition, $\sum_{n=0}^{N}P\left(n\right)=1\Rightarrow \sum_{n=0}^{5}P\left(n\right)=\left(7.33\right)P\left(0\right)\;$

⇒ P(0) = 0.136 #(16)

And from Equations (5)–(9), the system metrics can be computed sequentially as follows:

(17) $$L_{s}=\sum_{n=0}^{N}n\;P\left(n\right)=P\left(1\right)+2P\left(2\right)+3P\left(3\right)+4P\left(4\right)+5P\left(5\right)=\left(14.333\right)\cdot \left(0.136\right)=1.949\;$$

(18) $$L_{q}=\sum_{n=R}^{N}\left(n-R\right)\;P\left(n\right)=\sum_{n=4}^{5}\left(n-4\right)P\left(n\right)=P\left(5\right)=0.0453\;$$

(19) $$E\left[I\right]=\sum_{n=0}^{R-1}\left(R-n\right)P\left(n\right)=\sum_{n=0}^{3}\left(4-n\right)P\left(n\right)=4P\left(0\right)+3P\left(1\right)+2P\left(2\right)+P\left(3\right)=2.085\;$$

E[B] = R − E[I] = 4 − 2.085 = 1.915(20)

(21) $$P_{B}=\sum_{n=R}^{N}P\left(n\right)=\sum_{n=4}^{5}P\left(n\right)=P\left(4\right)+P\left(5\right)=0.136\;$$

Ws = Ls/λ = 1.949/2 = 0.9745 and Wq = Lq/λ = 0.0453/2 = 0.0223(22)

**Figure 5:**
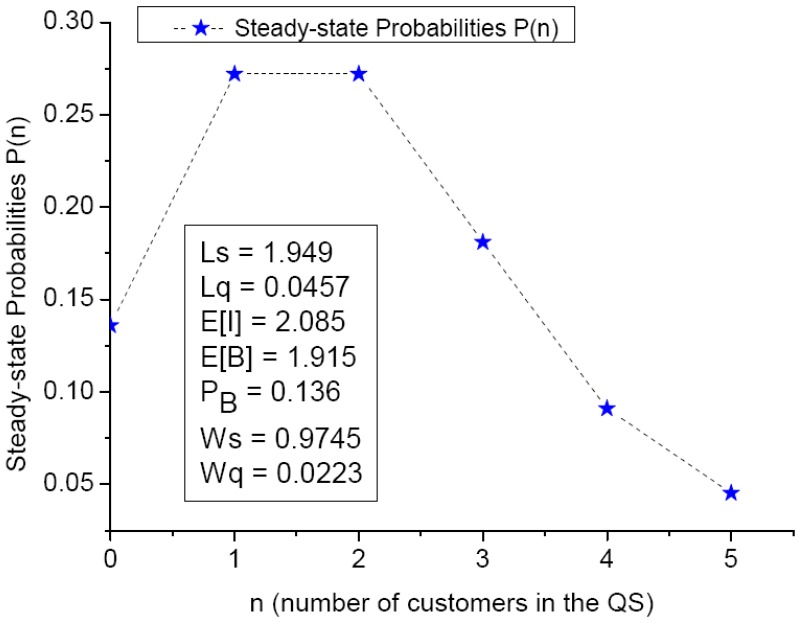
Steady-state probabilities with parameters (R, N, λ, μ) = (4, 5, 2, 1).

The distribution of steady-state probabilities is depicted in Figure 5. The relevant system performance measures, such as Ls, Lq, E[B] and Ws, are shown in the left-lower part of Figure 5. The average number of customers in the QS and the queue buffer are 1.949 and 0.0457, respectively. The average waiting times in the system and the queue buffer are 0.9745 and 0.0223, respectively.

## 5. Issue on Decision Support for HED Management

### 5.1. Evaluation Formulation on Cost

The strategy to minimize the total cost of the operating horizon is referred to as the optimal policy. Like all medical institutions, the cost pattern is important for gaining long-term and stable hospital management. To optimize the cost, we developed a steady-state expected cost function per unit time for an M/M/R/N queuing system, in which the parameter vector of (R, N, λ, μ) and the average waiting times (Lq) are considered as decision variables. The cost element C_W_ is defined as the waiting cost per unit time (or cost rate) per customer (HED patient) present in the system. Our goal is to provide decision support for determining the optimal number of servers R, say R*, to optimize the cost function. To formulate the cost function, some cost parameters are defined in the following vector form as follows:

Cq = cost per unit time when one customer is waiting for service,
Cs = cost per unit time when one customer joins the system and is served,
(C_B_, C_I_) = cost per unit time when one server is (busy, idle).

Using the definitions of each cost element with its corresponding feature, the cost function F(R, N) can be developed in association with the system metrics Ls, P_B_, Lq, E[I], and E[B], which are given in Equations (10)–(12), (7) and (8), respectively. It is noted that the steady-state probabilities for two segments are given in Equations (2) and (3). The probability that there is no customer in the system, P(0), is given by Equation (4).

(23) $$\begin{array}{c} F(R,\;N)=\mathrm{Cq}\;\mathrm{Lq}+\mathrm{Cs}\;\left(\mathrm{Ls}-\mathrm{Lq}\right)+C_{B}\;E[B]+C_{I}\;E[I]=\left(\mathrm{Cq}-\mathrm{Cs}\right)\;\mathrm{Lq}+\mathrm{Cs}\;\mathrm{Ls}+C_{B}\;E[B]+C_{I}\;E[I]\\ =\left(\mathrm{Cq}-\mathrm{Cs}\right)\;\frac{\rho^{R}P\left(0\right)}{R!}\sum_{j=0}^{N-R}\;\left[j\cdot {\left(\rho_{s}\right)}^{j}\right]+\mathrm{Cs}\;\{\sum_{n=0}^{R-1}n\cdot \frac{\rho^{n}}{n!}P\left(0\right)+\frac{\rho^{R}P\left(0\right)}{R!}\sum_{j=0}^{N-R}\;\left[j\cdot {\left(\rho_{s}\right)}^{j}\right]+R\cdot \\ \frac{\rho^{R}}{R!}\frac{\left[1-{\left(\rho_{s}\right)}^{N-R+1}\right]}{\left(1-\rho_{s}\right)}\}+C_{B}\;(R-\sum_{n=0}^{R-1}\;\left(R-n\right)\;P\left(n\right))+C_{I}\;[\sum_{n=0}^{R-1}\;\left(R-n\right)\;P\left(n\right)] \end{array}$$

The cost function F(R, N) in Equation (23) is expressed in terms of basic parameters, such as (R, N, λ, μ), and cost elements. It is noted that the utilization parameters of the unit server and system is given by (ρ, ρ_S_) =(λ/µ, λ/(Rµ)), respectively. The state probability functions P(n) for two segments are given in Equations (2) and (3), which are quite complex for the control parameter R. To find the optimal profile on the cost function, it is necessary to show the existence of convexity or unimodality of F(R, N). However, this mathematical task is difficult to implement. The cost function F(R, N) is unimodal; that is, it has a single relative minimum.

### 5.2. Evaluation of Cost Optimization

Equation (23) shows that the parameter R occurs not only at the location of in-line items, but also at the upper limit of the summation symbol Σ, which makes F(R, N) a highly nonlinear and complex function. Instead, practical numerical examples are presented and intensively studied by applying the proposed models. The optimization evaluation is firstly probed in terms of cost patterns in this subsection. For illustrative purposes, we first study the effect of varying R while keeping N constant, and then varying N while keeping R constant. All simulations are performed with the MATLAB platform with custom MATLAB scripts. The exemplified system parameters are listed as vector forms as follows:

(a) Average arrival rate of patients (λ) = 2.5, 3.0, and 3.5,
(b) Average service rate of a server (µ) = 1,
(c) Cost rate: (Cq, Cs, C_B_, C_I_,) = (200, 150, 120, 100),
(d) N = 15 for emergency departments of small and medium size.

**Figure 6:**
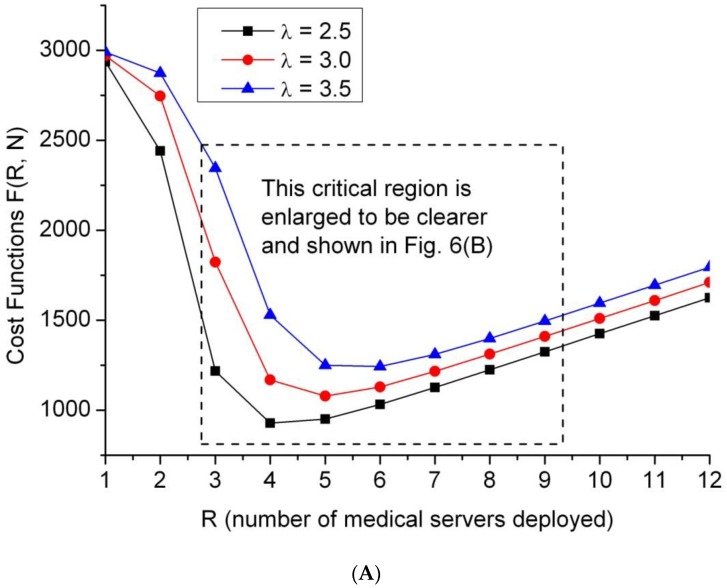

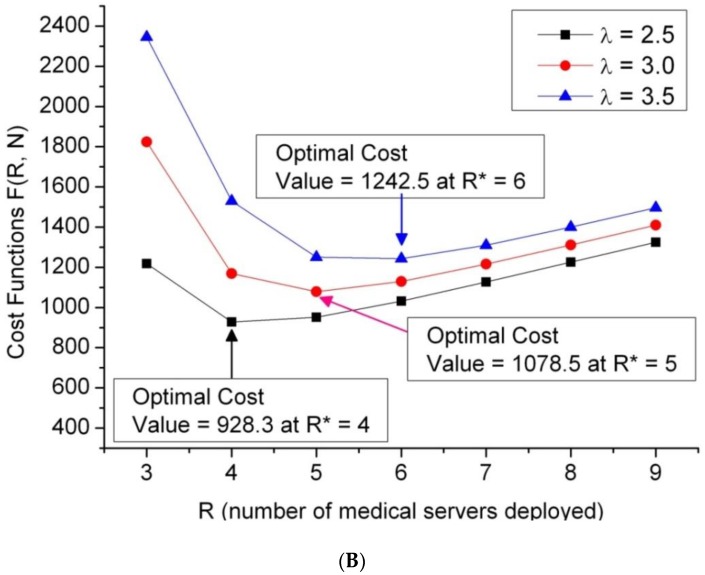
(**A**). Optimal cost patterns shown in terms of three average arrival rates. (**B**) An enlarged diagram showing the optimal cost data from Figure 6A.

Contour plots may provide the best graphical representation of the optimization problem, and also possess a powerful visualization that permits the solutions of the optimization problem by inspection. To validate the analytical solution, the graphical results were obtained and are shown in Figure 6A, where three cost contours with the black box, red circle, and blue triangle icons are depicted along the Y-axis in terms of λ = 2.5, 3.0, and 3.5, respectively. Generally, a higher patient arrival rate implies that the medical service cost is higher, so the blue line marked with the triangle icon (λ = 3.5) is situated over the red line marked with a circle (λ = 3.0). To clearly show the crucial region surrounded by the dashed-line rectangle in Figure 6A, enlarged detail is depicted in Figure 6B. In Figure 6B, the critical region is between R = 3 and R = 9 on the X-axis. The optimal cost value with the corresponding optimal R* is shown for each contour.

### 5.3. Issues on Cost Profile under the Constraint of Average Waiting Time

In view of performance evaluation, the average waiting time (AWT) can also be regarded as a measure of performance committed to the HED patients, and of a yardstick for comparing the effectiveness of the deployment of the staffing providers in a quantitative manner. Practically, it is reasonable for management experts to guarantee an AWT target level when they want to alleviate the sense of worry for potential HED patients. Logically, the higher the number of staffing providers deployed, the higher the cost. Hence, the proposed approach explores the issue of decision support for optimal cost under the constraint of the AWT at some target level.

**Figure 7:**
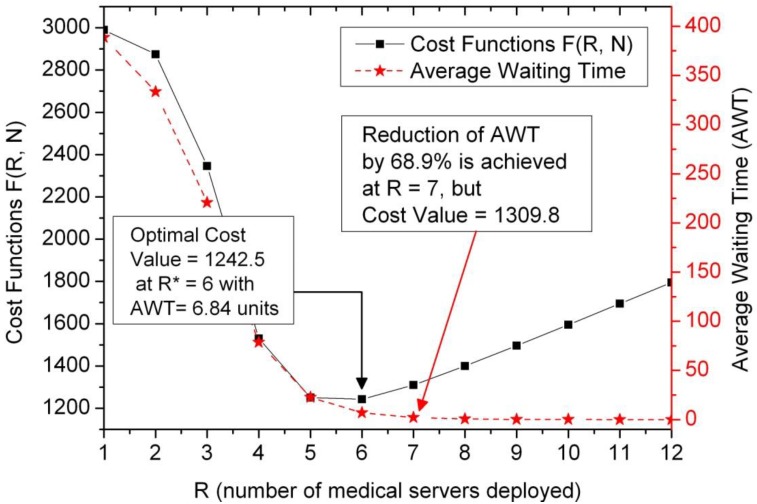
Decision support on optimal cost at R* = 7 under the constraint of reduction of AWT (average waiting time) by 68.9%, which is calculated from ((6.84–2.13)/6.84) × 100%.

In Figure 7 with the double-Y axis, the left Y-axis and the right Y-axis are set to be the cost values and the average waiting time (AWT), respectively. Observing the solid-line contour marked with black rectangles (i.e., the left Y-axis), the optimal cost value F(R, N) = 1242.5, which occurs at R* = 6 based on the similar parameters in Figure 6A with the average arrival rate λ = 3.5. However, the corresponding AWT approaches 6.84 units, which is a reference metric for decision-making. The proposed generic model could be used for general insights into the issues faced in deploying multiple staffing providers for a disease chain or a single department like the Department of Pediatrics shown in the middle right-handed location of the ground floor in Figure 1. On the right Y-axis, the dash-curve marked with a red star shows the variation profile of the performance metric for AWT.

During the busy time-window for a specific disease chain in HED, patients may spend hours in crowded waiting rooms before seeing a doctor. Those who choose to tolerate longer waiting times expose themselves to others who may have a contagious illness. To alleviate such an occasional impact, one straight approach for reducing the waiting time is to deploy more staffing providers for that specific disease chain. The simulation results in Figure 7 provide an exemplified decision reference on re-deploying the amount of staffing providers to alleviate the waiting time.

Then an issue emerges from the judgment: how many extra staffing providers are needed to gain a reduction in the AWT by some level (for example, 50%) without over-provisioning? Observing the red-star contour with the right Y-axis in Figure 7, it is found that the AWT can be reduced by 68.9% at R* = 7 (shown by the red dash-line) at the expense of only adding one staffing provider and cost values F(R* = 7, N) = 1309.8, compared to the minimum cost F(R = 6, N) = 1242.5. The detailed numerical data are listed in Table 1 with a range of R from unity to 12. The value of AWT for R = 12 is less than 0.01 and then marked to be 0 for clarity. In other words, the proposed approach can provide a quantitative decision support on the trade-off study between the cost profile and the amount of staffing providers in HED deployment.

### 5.4. Application Profile in a Window-by-Window Way

**Figure 8:**
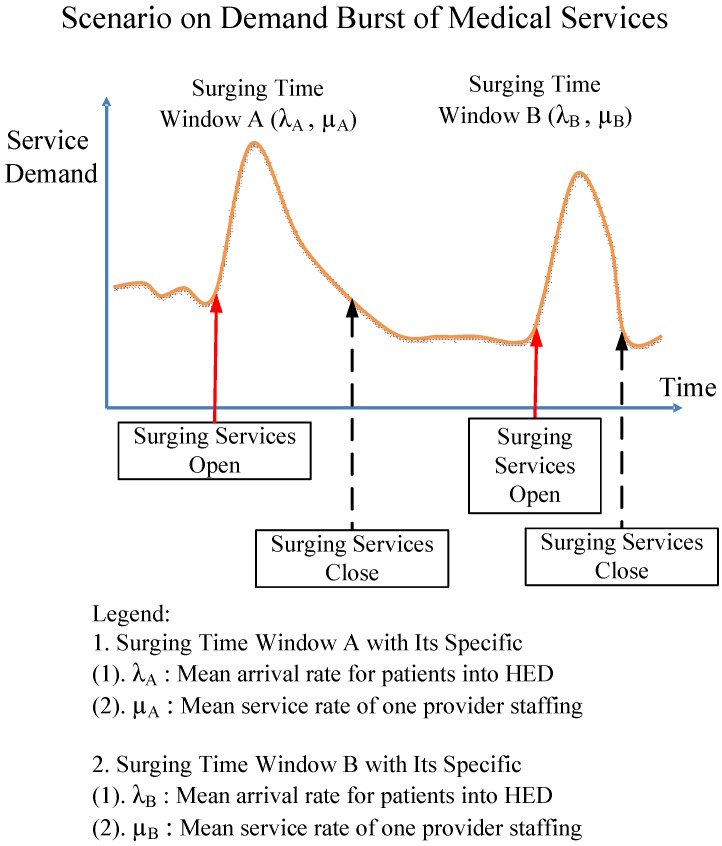
Iterative applications exemplified by various time-windows.

This work addressed the issue of the mathematical modelling to evaluate scenarios for deployment of medical resources to the HED, and also aimed to provide feasible applications iteratively to approaching an effective decision support in terms of deploying appropriate staffing providers to alleviate the impact on HED crowding. Patients who want to receive medical services always arrive randomly, and they require immediate services available at that time. If the service facility is operating at peak capacity when they arrive, they are obliged to wait in line (queue) with patience in the case of a shortage in staffing level. The surges and changes in HED activity may occur from time to time in terms of various time-widows with associated system parameters, as shown in Figure 8.

In Figure 8, each unique time-window may represent a specific surge time-period, wherein larger numbers of patients nearby the hospital are delivered to HED after some disaster or traffic event has occurred. In modeling language, the proposed modeling approaches can be applied in a window-by-window way that each specific time window can be approximated by its λ (average arrival rate) and µ (average service rate) in association with various practical historical data. For example, supposing that the surge in time window B of Figure 8 represents some middle-level traffic event, then the system parameters λ_B_ (average arrival rate) and µ_B_ (average service rate) may be approximated by some existing past and experienced parameters for the baseline, and then the cost function F(R, N) (23) may be applied iteratively to approach the cost optimization in a window-by-window way.

## 6. Conclusions

In terms of patient flow and available resources, an efficient generic methodology to optimize the performance of the HED platform has been addressed in this research. The proposed queue-based approach provides HED administrators with an efficient deployment of staffing providers to optimize the cost profile. Conceptually, the HED service platform was mapped into an M/M/R/N queuing system, and illustrated using appropriate figures and materials in the work. To gain insight into the queuing model, the mathematical derivation was detailed for the application need as well.

Based on the quantitative analysis, the M/M/R/N queue model was applied and derived, and then the relevant system metrics were established in a brand-new manner. The mathematical expression for cost function was established for evaluation requirements. In regards to verification, the relevant experimental results were obtained in terms of integration configurations on cost optimization and average waiting time. Instead of chaotic management, the proposed generic methodology may provide feasible applications for approaching an effective decision support in terms of deploying appropriate staffing providers to alleviate the impact on HED crowding.

## Figures and Tables

**Table 1:** Numerical data on AWT and the corresponding cost values for the range of R from unity to 12.

R	Cost Values	AWT	R	Cost Values	AWT
1	2990.0	388.57	7	1309.9	2.13
2	2873.9	333.42	8	1399.5	0.65
3	2345.8	220.77	9	1496.3	0.19
4	1530.2	78.57	10	1595.4	0.05
5	1250.7	22.51	11	1695.1	0.01
6	1242.5	6.84	12	1795.0	0

AWT, average waiting time.
